# 216. A Comparison of Multidrug-Resistant Gram-negative Bacteremia in Cancer and Non-Cancer Patients

**DOI:** 10.1093/ofid/ofad500.289

**Published:** 2023-11-27

**Authors:** Muhammad Haris Khan, Ruhul Munshi, Tamna Wangjam, Mohammad Alfrad Nobel Bhuiyan, Michelle Self, Mohammad Alam, Alexandre E Malek

**Affiliations:** LSU Health Shreveport, Shreveport, Louisiana; LSU Health Shreveport, Shreveport, Louisiana; LSU Health Shreveport, Shreveport, Louisiana; Louisiana State University Health Shreveport, Shreveport, Louisiana; LSU Health Shreveport, Shreveport, Louisiana; Louisiana State University Health Sciences Center, Shreveport, LA, USA, shreveport, Louisiana; LSU Health Shreveport, Shreveport, Louisiana

## Abstract

**Background:**

Multidrug-resistant gram-negative bacteremia (MDR-GNB) is a growing threat worldwide as it is associated with increased morbidity and mortality. We sought to describe the clinical features of MDR-GNB and outcomes in cancer and non-cancer Pts.

**Methods:**

We performed a retrospective study of adult patients with MDR-GNB at the Ochsner LSU Health Shreveport between January 2018 and July 2022. We collected patients' demographics, medical comorbidities, cancer diagnosis, causative bacteria (MDROs), source of bacteremia, antibiotics therapy, and clinical outcomes.

**Results:**

We identified 112 Pts with MDR-GNB. Pts were divided into 2 groups: cancer group (n=33, 29%) and non-cancer group (n=79, 71%). In the cancer group, the majority of Pts had solid tumors (n=20, 61%) vs liquid tumors (n=13, 39%). Mean age was 69 y in cancer compared to 57 y in the non-cancer group. Enterobacteriaceae were isolated in 28 cancer Pts (85%) including ESBL E. coli in 20 Pts (61%), Klebsiella s.p (CRE/ESBL) in 5 Pts (15%), and ESBL P. mirabilis in 3 Pts (9%). Pseudomonas aeruginosa CRO in 3 Pts (9%) and A. baumannii in 2 Pts (6%). In non-cancer Pts, Enterobacteriaceae were isolated in 65 Pts (82%) including ESBL E. coli in 40 Pts (51%), Klebsiella s.p (CRE/ESBL) in 18 Pts (23%) and ESBL P. mirabilis in 6 Pts (8%). P. aeruginosa CRO found in 6 Pts (8%) and A. baumannii in 8 Pts (10%). The sources of bacteremia in cancer group were UTI in 14 Pts (42.4%), osteoarticular infection (OAI) in 5 Pts (15%), pneumonia in 3 Pts (9%), CLABSI in 2 Pts (6%), endovascular in 1 Pt (3%) and unknown in 8 Pts (24%), whereas in non-cancer group, UTI was reported in 40 Pts (68%), pneumonia in 9 (27%), OAI in 1 (1.3%), endocarditis in 1 Pt (1.3%), and unknown in 20 Pts (25.3%). Mean duration of targeted antibiotics was 9 days in cancer vs 13.3 days in non-cancer group (*p-value*=0.4). Mean length of hospital stay was comparable, 34 d in cancer vs 31 d in non-cancer(*p-value*= 0.87). 9 Pts were admitted to ICU in cancer group (27%) vs 39 Pts (49%). Mortality rate was 39% in cancer (8 pts went to hospice) vs 16.4% in non-cancer.

**Conclusion:**

Our data showed that the common source of MDR-GNB remains urinary tract infection in both groups and the bacterial epidemiology is comparable between the two groups. Further studies are warranted to assess the impact of resistant organisms on cancer Pts.

**Disclosures:**

**All Authors**: No reported disclosures

